# Hypoxic Preconditioning Mitigates Acute Hypoxia Induced MS/VDB Cholinergic Cell Loss and Memory Impairments

**DOI:** 10.14336/AD.2025.0175

**Published:** 2025-04-05

**Authors:** Linhui Qin, Yong Yang, Houdi Zhang, Sijie Li, Xi Wu, Minjie Wang, Simeng Liu, Heguan Fu, Xunming Ji, Cong Han, Changhong Ren

**Affiliations:** ^1^Beijing Key Laboratory of Hypoxia Translational Medicine, Xuanwu Hospital, Capital Medical University, Beijing, China.; ^2^Center of Stroke, Beijing Institute of Brain Disorder, Capital Medical University, Beijing, 100053, China.; ^3^Hypoxia Conditioning Translational Laboratory of Clinical Medicine, Capital Medical University, Beijing, China.; ^4^School of Chinese Medicine, Beijing University of Chines Medicine, Beijing 100029, China.; ^5^Department of Neurosurgery, the Fifth Medical Centre, Chinese PLA General Hospital, Beijing, China.; ^6^Chinese PLA Medical School, Chinese PLA general hospital, Beijing, China.; ^7^Department of Neurosurgery, the First Medical Centre, Chinese PLA General Hospital, Beijing, China.

**Keywords:** Acute Hypoxia, Cholinergic Cell, Hypoxic Preconditioning, Cognitive Dysfunction, Hippocampal Synaptic Plasticity

## Abstract

Cholinergic cells originating from the medial septal nucleus (MS) and the vertical and horizontal limbs of the diagonal band of Broca (VDB and HDB, respectively) are critical for supporting a variety of memory and cognitive functions. However, the viability of cholinergic cells has not been explored in the context of acute hypoxia (AH). This study aimed to investigate the effects of AH on cholinergic cells in these nuclei and to test whether hypoxic preconditioning (HPC)—a previously established neuroprotective therapy—could prevent cholinergic cell loss, cognitive dysfunction, and hippocampal synaptic dysfunction in mice exposed to AH. We found that cholinergic cell loss occurred in the MS/DB after AH. HPC prevented this effect and also improved AH-induced cognitive dysfunction and hippocampal synaptic dysfunction. Overall, our findings highlight the significant role of cholinergic cells in AH-induced memory impairments and suggest that the preservation of cholinergic cell viability may provide a mechanism by which HPC improves memory impairments and preserves the function of memory-processing brain structures after AH.

## INTRODUCTION

Acute hypoxic injury occurs in various clinical and environmental contexts, including high-altitude exposure, asphyxiation, shock, acute respiratory distress syndrome (ARDS), cardiac arrest, sepsis, and perioperative complications. The brain is highly vulnerable to hypoxia, and acute hypoxic events can lead to cognitive impairments that profoundly disrupt daily function [1-5]. Rapid ascent to high altitudes is a common scenario where acute hypoxia (AH) occurs, often resulting in declines in learning, memory, and cognitive capacities. For example, acute high-altitude exposure has been shown to impair attention and sustained focus [6]. Individuals ascending to 3700 meters experience reduced alertness [7], and soldiers stationed at a high altitude of 4111 meters for 21 days exhibit a decline in cognitive function [8]. Additionally, prolonged high-altitude exposure impairs verbal and spatial working memory [9]. However, the mechanisms by which AH affects advanced brain functions, particularly learning and memory skills, remain to be fully uncovered.

The hippocampus, a critical region for cognitive functions, receives significant inputs from the medial septal nucleus (MS) and the vertical limb of the diagonal band of Broca (VDB). These inputs are essential for maintaining normal cognitive function and facilitating complex neural processes involved in learning and memory [10-12]. The MS/VDB-hippocampal projections are crucial for hippocampal function and play a significant role in neural networks and memory acquisition activities [13, 14].

Acetylcholine, an important excitatory neurotransmitter in the central nervous system, is mainly produced in the basal forebrain (BF), which includes structures like the MS and Diagonal Band of Broca (DB) [15]. Cholinergic neurons in the BF project fibers to the hippocampus via the MS-hippocampal pathway, significantly modulating hippocampal function and cognitive functions [16]. This cholinergic signaling is crucial for the regulation of memory and the facilitation of network activities within the hippocampus [17, 18].

γ-Aminobutyric acid (GABA), the main inhibitory neurotransmitter in the brain, plays a crucial role in maintaining cognitive functions by balancing excitatory (cholinergic) inputs [19]. Hypoxia can disrupt this balance by altering GABA signaling, including the expression of GABA receptors and transporter proteins, leading to various neurological and neuropsychiatric disorders [20, 21].

Understanding how AH impacts these neuro-transmitter systems, and their associated neural pathways is the key to comprehend its effects on cognitive functions and underscores the importance of developing effective treatments like hypoxic preconditioning (HPC) to mitigate these impacts. HPC is a non-pharmacological treatment aimed at improving clinical outcomes through sub-lethal hypoxia exposure, which induces adaptive molecular and the cellular responses [22]. Recent studies have elucidated the novel mechanisms underlying HPC-mediated neuroprotection. Zhang et al. demonstrated that HPC increases the expression of brain-derived neurotrophic factor (BDNF) by inhibiting DNMTs and reducing BDNF gene methylation, thereby activating BDNF/TrkB signaling to enhance learning and memory [23]. Furthermore, HPC has been shown to regulate mitochondrial dynamics by promoting mitophagy through the PINK1/Parkin pathway, thereby maintaining neuronal energy homeostasis [24]. Emerging evidence also highlights the role of HPC in modulating neuroinflammation; a 2024 study revealed that HPC inhibits NLRP3 inflammasome activation, there reducing pro-inflammatory cytokine release in microglia [25]. These mechanisms collectively enhance cerebral hypoxia tolerance. Our laboratory has previously demonstrated that HPC improved neurogenesis in long-term hypoxia exposure model [26]. However, it remains unknown whether AH affects cholinergic signaling from the BF to the hippocampus, thereby impacting memory acquisition activities, and whether HPC can improve these effects.

The present study aimed to investigate whether AH exposure induces neuronal cell loss within BF nuclei and to examine the effects of HPC on cell survival in the BF, cognition, and synaptic function in structurally connected brain regions following hypoxic conditions. For this purpose, we used a C57BL/6 mouse model of AH, assessed cognitive and locomotor functions, evaluated cholinergic and GABAergic cell survival in the BF, hippocampus, and other nuclei, and measured synaptic plasticity. Additionally, we examined the protective effects of HPC. Our findings reveal for the first time that AH exposure leads to the loss of cholinergic cells within the MS/VDB. This important finding indicates that cognitive impairment is not caused by hippocampal damage. Notably, HPC was able to mitigate this effect, improving both memory and hippocampal function.

## MATERIALS AND METHODS

### Animals

Eight-week-old male C57BL/6 mice (20-25 g) were purchased from Vital River Laboratory Animal Technology Co. Ltd. They were housed in a temperature-controlled room (22 ± 2°C) with controlled humidity (45 ± 5%) and a 12-hour light-dark cycle [27]. All animal experiments in this study were reviewed and approved by the Institutional Animal Care and Use Committee of Capital Medical University on October 14, 2021 (date and number: 2021/255) and complied with the Guide for the Care and Use of Laboratory Animals. During the experiment, the body weight and health status of mice were monitored daily. Any animal exhibiting a weight loss exceeding 15% of initial body weight or severe distress (e.g., immobility, inability to eat/drink) would be euthanized in accordance with ethical guidelines and excluded from analysis. No mice met these criteria in the current study.

### Acute hypoxia model

**Figure 1.**
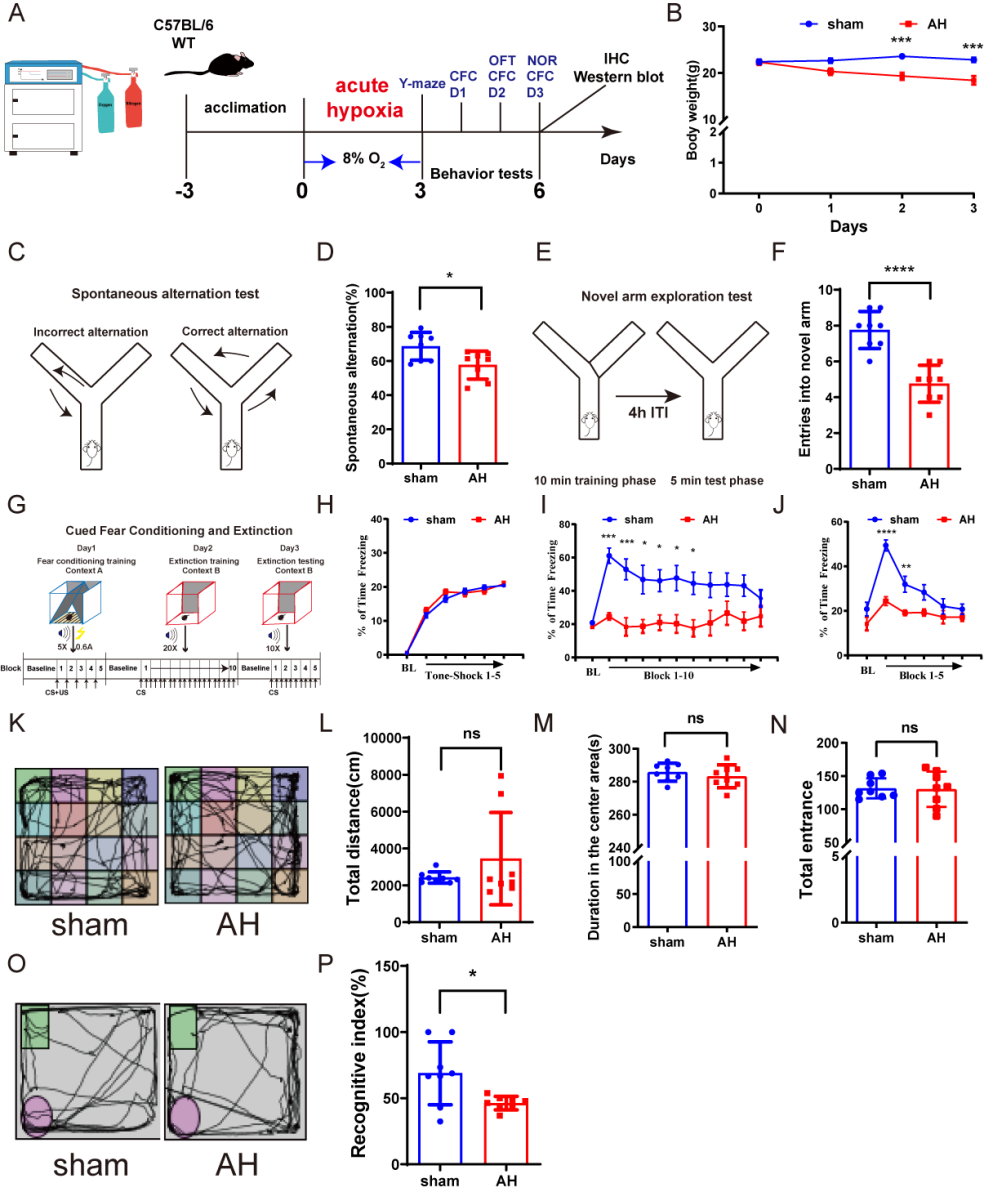
**AH induces cognitive dysfunction in mice**. (**A**) A schematic diagram of the hypoxic chamber and the AH experimental design. (**B**) Body weight changes in both groups of mice (n=8). AH: acute hypoxia exposure. (**C-F**) Y-maze test (n=8). (**C**) Schematic diagram of the spontaneous alternation experiment; (D) The spontaneous alternation rate; (E) Novel arm exploration diagram; (F) Entries into the new arm of both groups of mice. (**G-J**) Cued fear conditioning and extinction test (n=8). (**G**) Experimental design; (H-J) Freezing response measured on day 1 (H), day 2 (I), and day 3 (J). (**K-N**) Open Field Test (OFT) (n=8). (**K**) Representative activity tracking in the OFT; (L) Total distance; (M, N) Time and entrance in the center. (O, P) Novel Object Recognition (NOR) (n=8). (**O**) Representative activity tracking in the NOR; (P) The recognition index. Data are shown as mean±SD. Significant differences as determined by unpaired Student’s t-test (two-tailed). ns: no significant differences; * *p* < 0.05, *** *p* < 0.001, **** *p* < 0.0001, unpaired t-test and repeated-measures ANOVA analysis of variance.

The AH models were carried out in a hypoxic oxygen chamber with a stable and controlled rate by the gas control delivery system (ATTENDOR140PRO, Guangzhou Huayuexing Medical Technology Co.). The hypoxic environment was produced with different ratios of 99.99% oxygen and 99.99% nitrogen gases. The oxygen concentration in the chamber was automatically controlled. Mice were directly exposed to 8% O_2_ from a normoxic environment (21% O_2_) without a gradual transition. This rapid transition was controlled by the gas delivery system to simulate AH conditions. Mice were maintained in the hypoxic chamber at 8% O_2_ for 3 consecutive days (Fig. 1A), during which they had free access to food and water within the chamber. Control animals were individually housed under normoxic conditions with identical access to food and water [26].

### Hypoxic preconditioning

Hypoxic preconditioning protocol consisted of a 10-min normoxic (21% O_2_) followed by a 10-min hypoxia (10% O_2_), repeated for 5 consecutive cycles each day for 5 consecutive days. Control mice were placed in the same hypoxic chamber with continuous normoxic air circulation during equivalent time periods. Hypoxic preconditioning was initiated daily at 7:00 p.m. During non-hypoxic hours, the mice were fed in a standard normoxic conditions with free access to food and water [28].

### Mouse behavior tests

All behavioral analyses were conducted on adult mice, and experimental tests were scheduled within fixed time periods to minimize experimental variations. To maintain consistency in the experimental procedure, the behavioral tests were performed in a specific sequence. the behavioral assays were conducted in the following order: Y-maze test, cued fear conditioning and extinction, open field test and novel object recognition test. Additionally, the human observers were blinded to the group allocation.

#### Y-maze

Y-maze was a definitive test for examining the short term memory and spatial learning of mice [29]. The Y-maze spontaneous alternation test was performed as previously described to assess spontaneous alternation, which was defined as successive entries into the three arms in overlapping triplet sets [30]. The maze had three equal arms (35 cm long, 15 cm high, and 5 cm wide) converging at equal angles (Shanghai Xinruan Information Technology, Shanghai, China). Each mouse was placed in the center and allowed to explore for 5 minutes. The total number of arm entries and alternations were counted, with the alternation rate calculated as [number of correct alternations/(total entries - 2)] × 100% [31].

The Y-maze novel arm exploration test included three arms: a start arm (always open), a novel arm (blocked during the first trial), and another arm (always open). During the first 10-minute trial, the mouse explored the start and other arms, while the novel arm was blocked. After a 4-hour inter-trial interval (ITI), the second 5-minute trial allowed the mouse free access to all arms, including the novel arm, to assess memory and preference for the novel arm [32]. Total arm entries were recorded and analyzed to control potential locomotor activity differences.

#### Cued fear conditioning and extinction

Cued fear conditioning (CFC)—a form of Pavlovian conditioning—was used to assess associative fear memory, extinction, and extinction retention [33]. CFC and extinction training/testing consisted of three days. On the first day, mice were placed into a triangular cage with a shock grid floor. During this first session, animals were exposed to the training context (Context A), which was illuminated by yellow light and had an acetic acid smell. After allowing animals to explore the cage for 3 minutes, an auditory cue (tone, 4000 Hz, 80 dB) sounded for 10 seconds, which co-terminated with a 1-second-long footshock (0.6 mA), and was repeated 5 times separated by a 60-second intertrial interval (ITI). On day 2, animals were returned to the testing room and placed in the square cage that now had a soft floor, red light, and vanilla scent (Context B). Animals were allowed to explore the cage for 3 minutes, after which a 10-second-long tone (4000 Hz, 80 dB) was presented 20 times (60-second ITI). On day 3, animals were exposed again to Context B and a 10-second-long tone was presented after 3 minutes for a total of 10 times (60-second ITI). Percentage of time freezing was quantified using Freeze Frame software (Labmaze Conditioned Fear Video Analyser, Beijing Zhongshi Dichuang Technology Development Co., Ltd)) during the first 3 minutes before tone presentation on each day (baseline measurement) and in 140-second blocks consisting of two trials (tone presentations) on days 2 (10 block total) and 3 (5 blocks total). The behavioral apparatus was cleaned thoroughly with 70% ethanol after each animal.

#### Open field test

The open field was used to evaluate anxiety-like behaviors and locomotor function. The spontaneous activity of animals was measured by open field experiment. The distance of movement, entrance and duration in the center area of mice were recorded within 5 min. The behavioral apparatus was cleaned thoroughly with 70% ethanol after each animal [34].

#### Novel object recognition

The novel object recognition test is used to evaluate object recognition memory through three phases [35]. For three phases, adaptation period: each mouse was placed in an open testing chamber (40 cm x 40 cm) and allowed to explore for 10 min to adapt to the environment. Familiarisation period: two identical objects (A and B, make sure they were not pushed) of the same colour and shape and without smell were placed in the chamber, the identical objects are 10 cm from the side walls; mice were placed in the chamber with their faces towards the walls and their backs towards the objects from the middle of the two objects and were allowed to explore freely for 5 min and then the mice were taken out. Mice were tested after 1 hour of rest. Testing period: one of the two identical objects were replaced by an object of different shape and colour in the experimental box (A C or B C, making sure that the object had no smell and was not pushed), and the two different objects were both 10 cm away from the side wall; the mice were placed in the experimental box facing the wall of the box with their backs towards the objects from the middle of the two objects, and the activities of the mice were recorded over a period of 5 min. Novel object recognition was defined as spending more time sniffing new object (N) than familiar object (F). A mouse's proximity to an object was considered if its nose was within 2 cm. The recognition index was calculated as [(N)/(N + F)*100%], with a positive RI representing a preference for novel objects. Recognition memory was scored using each mouse RI [28].

### Electrophysiological recording

A multi electrode dish (MED64 planar microelectrodes; Panasonic, Osaka, Japan) was used to record field excitatory postsynaptic potentials (fEPSP). The fEPSP evoked at Schaffer collateral pathway CA1 synapses were recorded from the dendritic layer of CA1 neurons. The evoked fEPSP were amplified using a 64-channel amplifier and digitized at a sampling rate of 20 kHz. The input-output curve was obtained with an incremental stimulation intensity from 10 to 90 μA, and the stimulation intensity of subsequent fEPSP recordings were determined by the 30-50% maximum amplitude of the input-output curve. The stable baseline of fEPSP was recorded for 15 min, and traces were obtained and analyzed using the MED64 System software program. Long term potentiation (LTP) was induced by theta-burst stimulation (TBS), which consisted of five bursts at intervals of 10 s. Each burst contained four pulses at 100 Hz, with an inter-burst interval of 200 ms [36].

### Western blot analysis

Mice were anesthetized via intraperitoneal injection of 2% pentobarbital sodium (40 mg/kg), perfused with 0.9% sodium chloride, decapitated, and their brains were rapidly frozen in liquid nitrogen. Nuclei (nucleus accumbens, MS+DB, nucleus basalis of Meynert, hippocampus, amygdala, and cortex) were extracted using a brain punch (57402, Electron Microscopy Sciences). Equal amounts of protein and protein ladders were separated on SDS-PAGE gels and transferred to a PVDF membrane. Membranes were then blocked with 10% skimmed milk at room temperature for 1 h, Next, they were incubated with the primary antibody overnight at 4°C. The main primary antibodies used included choline acetyltransferase (ChAT, ab181023, Abcam, 1:1000), glutamate decarboxylase 67 (GAD67, GTX101881, GeneTex, 1:1000), vesicular GABA transporter (VGAT, GTX637107, GeneTex, 1:1000), BDNF (ab108319, Abcam, 1:1000,) and β-actin (TA-09, Zhongshan Golgen Bridge, 1:2000). After washing three times with TBST, the membranes were incubated with suitable secondary antibodies (1:2000, Zhongshan Golgen Bridge) at room temperature for 1 h. Protein bands were detected using an enhanced chemiluminescence kit (Thermo Fisher Scientific) and quantified with ImageJ software. Protein expression levels were normalized to β-actin, and the relative expression was calculated as the ratio of the target protein band intensity to the β-actin band intensity.

### Immunofluorescence staining

Mice were anesthetized intraperitoneally with 2 % pentobarbital sodium (40 mg/kg). The mice brain tissues were perfused with 0.9% sodium chloride and 4% paraformaldehyde (PFA). Immunofluorescence was performed on 20μm thick coronal brain slices. Brain sections were fixed in methanol, and membranes were ruptured with 0.25% Triton-X100. After blocking with 1% BSA, they were incubated with the primary antibody overnight at 4°C and then washed three times with PBS. A suitable secondary antibody (1:300, Invitrogen) was selected and incubated at room temperature for 1 h. The main primary antibodies used included: ChAT (ab181023, Abcam, 1:1000), GAD67 (GTX101881, GeneTex, 1:1000), NeuN (MAB377, Millipore 1:300). To confirm the specificity of the positive signals in our immunostaining experiments, we employed a negative control where only the secondary antibody was applied without the primary antibody. For each group of mice, three consistent regions were selected for calculation in each mouse.

### Golgi staining

Golgi staining was performed with the FD Rapid Golgi Stain Kit (PK401A, FD Neuro Technologie). Liquids A and B were mixed in a 1:1 ratio at least 24 h prior to use. Mice were anesthetized intraperitoneally with 2% pentobarbital sodium (40 mg/kg) and perfused with 0.9% sodium chloride. The hippocampal CA1 tissue samples were dissected and cut into blocks of 0.5-1 cm thickness. The brain blocks were immersed in a mixture of solutions A and B, which were replaced the next day, and incubated in the dark at room temperature for 2 weeks. The blocks were then transferred to solution C, which was replaced after 24 h in the dark for 4 days. The quick-frozen brain blocks were cut into 100 μm thick slices.

For the spine density analysis, images of dendritic spines from the secondary and tertiary branches of apical dendrites of hippocampal CA1 pyramidal neurons were captured under 1000 × magnification. Spine density was calculated by quantifying the number of spines per 10 μm of dendrite length [37]. The dendrite complexity of each pyramidal neuron was analyzed using the Sholl analysis described above [38]. Capture of CA1 pyramidal neurons from the hippocampus at 1000 × magnification.

### Imaging and cell counting

Immuno-positive cells were visualized and counted per field using a microscope with a motorized stage and Leica image-analysis system software. Briefly, three consecutive coronal sections per animal were digitized at low power (40x). Then every region of interest (MS, HDB, and VDB) of each hemisphere was outlined and 50% of the defined region of interest was randomly sampled. Immuno-positive cells were counted under 200× magnification.

Representative images were selected based on the following criteria: (1) Images were randomly captured from multiple independent biological replicates (n ≥ 3 per group) to avoid selection bias. (2) For immunofluorescence and Golgi staining, fields of view were systematically selected using a motorized stage (Leica) to cover the entire region of interest (e.g., MS/VDB or hippocampal CA1). (3) Selected images were required to align with group-level quantitative data (e.g., cell counts, fluorescence intensity) within one standard deviation of the mean. (4) All images were independently reviewed by two researchers blinded to experimental conditions to ensure objectivity.

### Statistical analysis

All data were expressed as means ± standard deviation (SD) and analyzed using GraphPad Prism (9.4.1) software. Data were analyzed using two-way repeated-measures ANOVA with time (within-subject factor) and group (between-subject factor) for longitudinal behavioral tests (e.g., fear extinction). For between-group comparisons at specific time points, unpaired Student’s t-tests were applied. All post-hoc analyses used Tukey’s HSD correction for multiple comparisons. Effect sizes were reported as partial eta-squared (η²) for ANOVA and Cohen’s d for t-tests. Statistical significance was set at *p*< 0.05. All data collection and analyses were performed by researchers blinded to experimental groups to ensure objectivity.

## RESULTS

### AH induces memory impairments in mice

To simulate AH, mice were exposed to 8% O_2_ for 3 consecutive days (Fig. 1A). Initial body weights of the mice were similar before AH exposure; however, after 3 days, the AH group exhibited a significantly reduced body weight compared to the sham group (Fig. 1B). Various behavioral tests were employed to assess cognitive and motor functions in the mice. The open field test was used to evaluate locomotor activity, while the Y-maze, cued fear conditioning and extinction, and novel object recognition tests were used to assess cognitive behavior.

In the Y-maze spontaneous alternation test (Fig. 1C), no significant difference was observed in total arm entries between the sham (33 ± 4.33) and AH groups (29.4 ± 2.5, P = 0.0756). However, the AH group exhibited a markedly reduced spontaneous alternation rate (Fig. 1D). In the Y-maze novel arm exploration test (Fig. 1E), the total entries remained comparable between groups (sham: 30.875 ± 3.219, vs. AH: 29.5 ± 4.243, P = 0.5057), while the percentage of entries into the new arm was significantly decreased in the AH group compared to the sham group (Fig. 1F) (*p* < 0.0001).

Previous studies have shown that lesioning MS cholinergic cells impairs the acquisition of cued fear and extinction (Fig. 1G) [39]. Baseline freezing levels were comparable between groups on Days 1 and 2 (Figs. 1H, I), as well as after the five conditioning shocks on Day 1. After presentation of the first tone on Day 2, there was a significant increase in the percentage of time freezing within the sham group, indicative that animals had acquired cued fear. However, mice in the AH group failed to show significant variation in freezing levels after the first tone. Within-group comparisons revealed that the sham group exhibited a significant increase in freezing levels on Day 3 compared to baseline, followed by a progressive decline across trials, indicating successful fear extinction. In contrast, the AH group showed no significant temporal changes in freezing. Between-group comparisons further demonstrated that the AH group had significantly lower freezing levels than the sham group during the first trial of Day 3 (Fig. 1J). These results collectively indicate that AH disrupts both the acquisition and retention of fear extinction.

In the open field test, no significant difference was observed in the total distance traveled (Fig. 1L), the duration in the center area (Fig. 1M), or the number of entrance (Fig. 1N) between the two groups, suggesting that spontaneous activity was not affected by AH.

**Figure 2.**
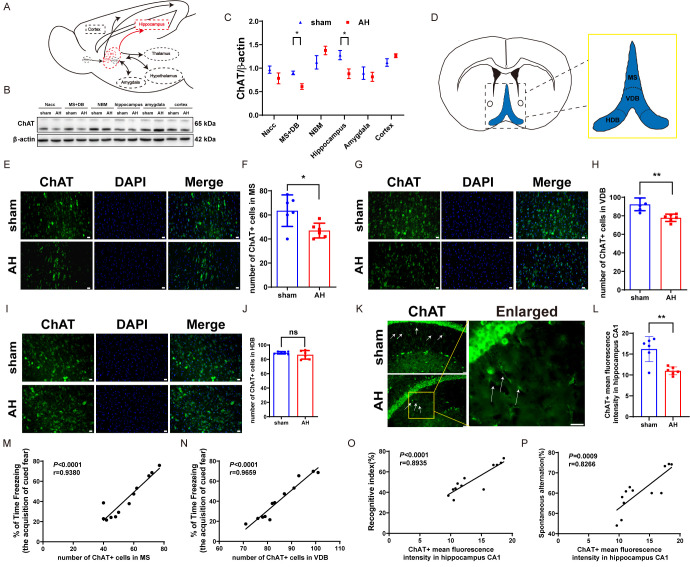
**AH induces cholinergic neuronal cell loss in the MS and VDB of mice**. (**A**) Schematic illustrating the main connections of the septal network in the rodent brain (sagittal view). (B, C) Western blot analysis of ChAT expression in various nuclei, including representative blot images and quantitative analysis (n=5). (**D**) Schematic outlining delimitations of the septal subregions for counting ChAT-immunopositive cells at Bregma +0.74. (E, F) Representative images and quantified number of ChAT-immunopositive cells per frame in the MS (n=6). (G, H) Representative images and quantified number of ChAT-immunopositive cells per frame in the VDB (n=6). (I, J) Representative images and quantified number of ChAT-immunopositive cells per frame in the HDB (n=6). (K, L) Representative images and quantified ChAT-immunopositive mean fluorescence intensity (showing cholinergic fibers (arrow heads) per frame in the hippocampus CA1 (n=6). (**M**) Number of ChAT-immunopositive cells in the MS positively correlated with freezing time after the first tone presentation on Day 2 of the cued fear conditioning test, as indicated by Pearson correlation analysis (n=6). (**N**) Number of ChAT-immunopositive cells in the VDB positively correlated with freezing time after the first tone presentation on Day 2 of the cued fear conditioning test, as indicated by Pearson correlation analysis (n=6). (**O**) Number of ChAT-immunopositive means fluorescence intensity in the hippocampus CA1 positively correlated with recognitive index, as indicated by Pearson correlation analysis (n=6). (**P**) Number of ChAT-immunopositive means fluorescence intensity in the hippocampus CA1 positively correlated with spontaneous alternation as indicated by Pearson correlation analysis (n=6). Data are shown as mean±SD. Significant differences as determined by unpaired Student’s t-test (two-tailed). ns: no significant differences; * *p* < 0.05, ** *p* < 0.01, unpaired t-test and Pearson correlation. Scale bars, 20 µm.

The novel object recognition test was used to assess the working memory. Compared with the sham group, the RI of the mice in the AH group was significantly reduced (Fig. 1O, P) (*p*<0.05). This result suggests that 3-day AH exposure reduces novel object exploration time and impairs cognitive function in mice.

Overall, the results suggest that 3-days AH exposure impairs cognitive functions related to the medial septum (MS) and hippocampus. However, since the cognitive tests used (e.g., Y-maze, novel object recognition, and fear extinction) also engage multiple cortical areas, including the prefrontal and retrosplenial cortices, it is possible that AH may also affect cortex-dependent functions [40]. For instance, the prefrontal cortex is critical for executive functions and fear extinction consolidation [41], while the retrosplenial cortex contributes to spatial memory integration and navigation [42].

**Figure 3.**
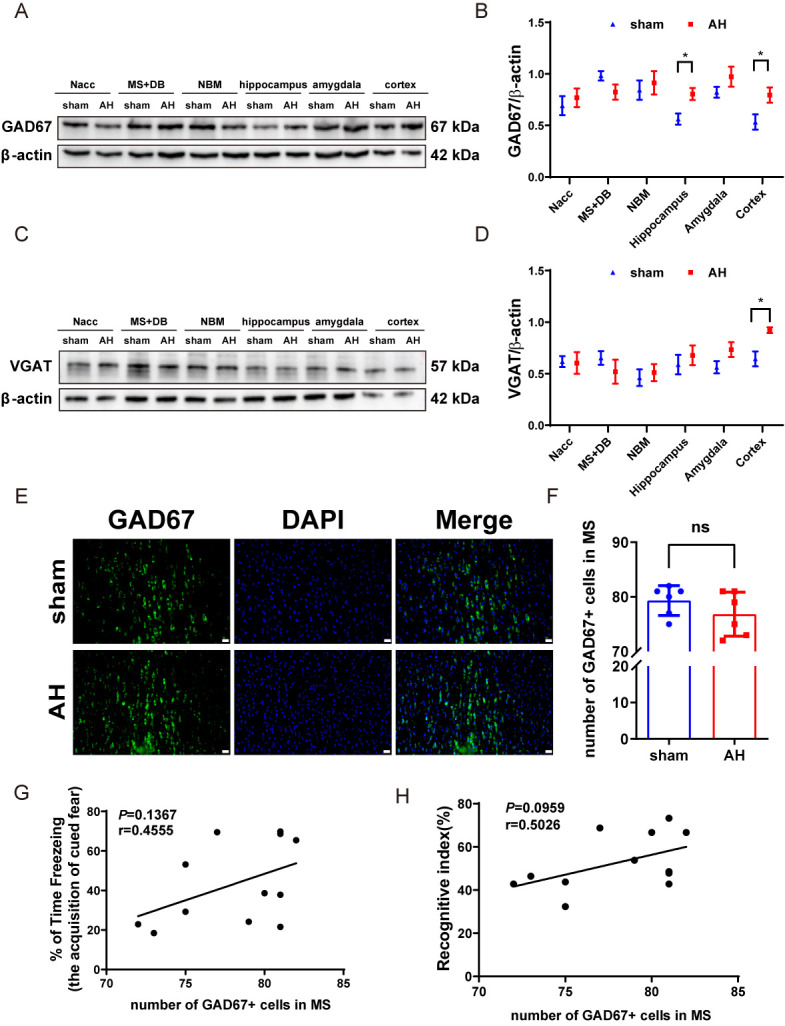
**AH shows no significant effect on MS GABAergic neurons in mice**. (A, B) Western blot analysis of GAD67 expression in various nuclei, including representative blot images and quantitative analysis (n=5). (C, D) Western blot analysis of VGAT expression in various nuclei, including representative western blot images and quantitative analysis (n=5). (E, F) Representative images and quantified number of GAD67-immunopositive cells per frame in the MS (n=6). (**G**) Correlations between the number of GAD67- immunopositive cells in MS and freezing time after the first tone presentation on Day 2 of the cued fear conditioning test (n=6). (**H**) Correlations between the number of GAD67- immunopositive cells in MS and recognitive index (n=6). Significant differences as determined by unpaired Student’s t-test (two-tailed). ns: no significant differences; *P < 0.05, unpaired t-test and Pearson correlation. Scale bars, 20 µm.

Previous studies have demonstrated that AH disrupts functional connectivity between the hippocampus and cortical regions, further exacerbating cognitive deficits [4]. Further studies are needed to specifically evaluate the impact of AH on distinct cortical regions.

### AH induces cholinergic cell damage in the MS/VDB of mice

The medial septum/diagonal band of Broca (MS/DB) is the primary source of cholinergic innervation to the cerebral cortex, hippocampus, and amygdala (Fig. 2A), and plays a crucial role in regulating neural circuits involved in learning and memory [43]. Previous studies have shown that MS/DB cholinergic neurons are vulnerable to various forms of hypoxic insults, such as cardiac arrest [44], ischemic stroke [45], and chronic cerebral hypoperfusion [46]. However, the impact of AH on these neurons and their role in cognitive dysfunction remain underexplored. To investigate whether AH induces neuronal cell death in the brain, we examined the survival of cholinergic and GABAergic cell within specific subregions at 3 days post-AH exposure. Western blot analysis revealed that AH significantly reduced the expression of ChAT, (a cholinergic neuron marker), in the MS/DB and hippocampus (*P*<0.05) (Fig. 2B, C). Immunofluorescence confirmed a significant loss of cholinergic cells in the MS/VDB (Fig. 2E-H), and cholinergic fibres in the hippocampus CA1 were also significantly reduced (Fig. 2K-L). While there was no significant difference in cholinergic cells between the two groups of mice in HDB (Fig. 2I-J). Pearson correlation analysis revealed that the number of cholinergic cells in the MS (Fig. 2M) and VDB (Fig. 2N) positively correlated with freezing time after the first tone presentation on Day 2 of the cued fear conditioning test (r=0.9380, *P* < 0.001 and r=0.9659, *P* < 0.001, respectively), reflecting impaired fear acquisition in AH-exposed mice. Additionally, cholinergic fiber intensity in the hippocampal CA1 region showed a positive correlation with the RI in the novel object recognition test (r=0.8935, *P* < 0.001; Fig. 2O) and the spontaneous alternation rate in the Y-maze (r=0.8266, *P* = 0.0009; Fig. 2P). While no significant correlation was observed between CA1 cholinergic fiber intensity and entries into the novel arm in the Y-maze (data not shown). Furthermore, the loss of cholinergic cells is associated with cognitive impairments, indicating that the viability of these cells is crucial for maintaining cognitive functions.

In addition to cholinergic cell loss, Western blot analysis showed that AH significantly upregulated the GABAergic neuron marker GAD67 in the hippocampus and cortex regions (Fig. 3A, B) (*P*<0.05), and another marker VGAT increased in the cortex (Fig. 3C, D). However, immunofluorescence staining revealed no significant difference in GAD67-positive cells between the AH-treated group and the sham group in the MS (Fig. 3E-F). Pearson correlation analysis revealed no significant correlation between the number of GABAergic cells in the MS and all behavioral tests, suggesting that AH does not affect GABAergic cells in the MS (Fig. 3G-H).

### AH induces hippocampal synaptic dysfunction.

**Figure 4.**
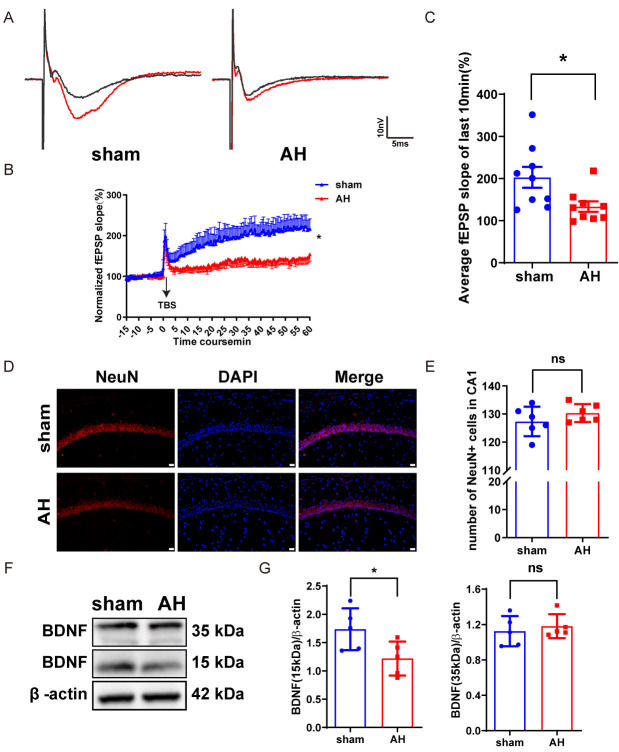
**AH impairs hippocampal synaptic function in mice**. (**A**) Representative traces of fEPSP before (black curve) and after (red curve) the stimulation of TBS in LTP induction. (**B**) Time-course changes in normalized fEPSP slopes (%), fEPSP were induced by TBS (n=9/6). (**C**) Quantitative analysis of average fEPSP slopes in the last 10 min of LTP (n=9/6). (D, E) Representative images and quantified number of NeuN- immunopositive cells per frame in the hippocampus CA1. (F, G) Western blot analysis of BDNF expression of hippocampus, including representative blot images and quantitative analysis (n=5). Significant differences as determined by unpaired Student’s t-test (two-tailed). ns: no significant differences; *P < 0.05, unpaired t-test and repeated-measures ANOVA analysis of variance.

Cholinergic neurons in the MS/DB region modulate neural activity in the cortex, hippocampus, and other brain areas through their axonal fiber projection. This neuromodulation may affect LTP in these circuits, thereby influencing learning and memory capabilities [47]. To investigate the impact of hypoxia on LTP, we performed electrophysiological recordings in the hippocampus of the mice. The results showed that the sham group successfully induced and maintained the LTP phenomenon. However, it was attenuated in AH mice (Fig. 4A-C). Neuronal death and loss are recognized as primary contributors to brain function impairment in the patients [48]. We assessed the expression of NeuN (a neuronal nuclei-specific marker) in hippocampal CA1 by immunofluorescence staining and found that there was no significance of neurons in hippocampal CA1 in AH mice (Fig. 4D, E). These findings suggest that AH induces impaired hippocampal synaptic function. To further understand the mechanisms underlying these impairments, we investigated the expression of BDNF, a key protein involved in synaptic plasticity and neuronal survival. Using a Western blot assay, we found that AH decreased the expression of BDNF (15kDa) compared to the sham group (Fig. 4F, G). BDNF is a well-established regulator of synaptic plasticity, particularly in the hippocampus, where it supports the induction and maintenance of LTP [49, 50].The reduction in BDNF expression observed in our study likely contributes to the impaired synaptic function and cognitive deficits seen in AH mice.

### HPC mitigates memory impairments in AH mice

**Figure 5.**
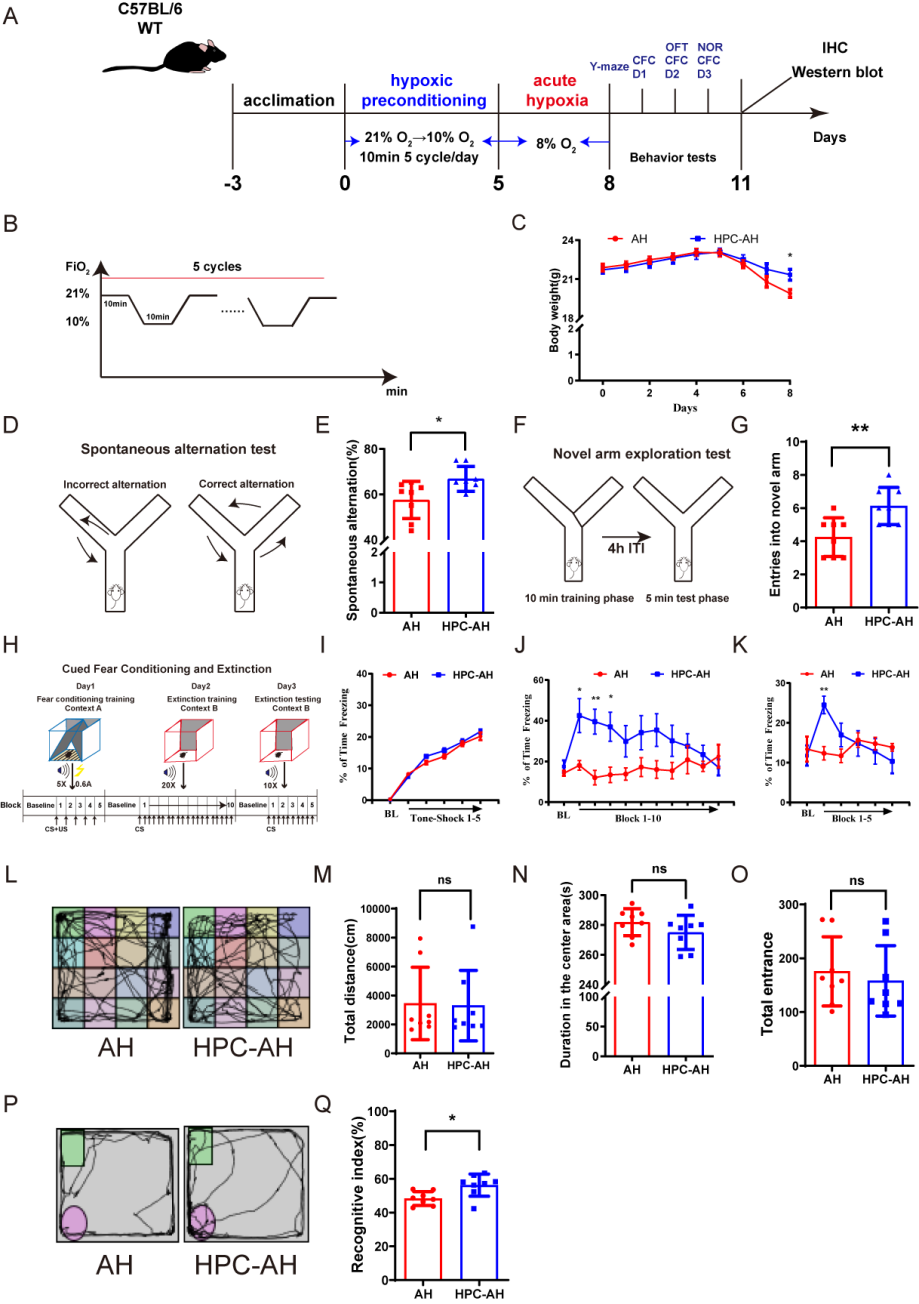
**HPC mitigates AH-induced memory impairments**. (**A**) HPC experimental design. (**B**) HPC model diagram (C) Body weight changes in both groups of mice (n=8). HPC-AH: AH mice preceded by hypoxic preconditioning. (**D-G**) Y-maze test (n=8). (**D**) Schematic diagram of the spontaneous alternation experiment; (E) The spontaneous alternation rate; (F) Novel arm exploration diagram; (G) Entries into the new arm of both groups of mice. (**H-K**) CFC and extinction test (n=8). (**H**) Experimental design; (I-K) Freezing response measured on day 1 (I), day 2 (J), and day 3 (K). (**L-O**) Open Field Test (OFT) (n=8). (**L**) Representative activity tracking in the OFT; (M) Total distance; (N, O) Time and entrance in the center. (P, Q) Novel Object Recognition (NOR) (n=8). (**P**) Representative activity tracking in the NOR; (Q) The recognition index. Data are shown as mean±SD. Significant differences as determined by unpaired Student’s t-test (two-tailed). ns: no significant differences; *P < 0.05, **P < 0.01, ***P < 0.001, unpaired t-test and repeated-measures ANOVA analysis of variance.

To investigate whether HPC can prevent cognitive dysfunction in AH exposure mice. The experimental design was shown in Fig. 5A, B. After 5 days of HPC, the body weight of mice in the HPC-AH group significantly increased compared to the AH group (*P*<0.001) (Fig. 5C).

In the Y-maze spontaneous alternation test (Fig. 5D), the total number of arm entries did not differ significantly between the AH group (29.375 ± 2.496) and the HPC-AH group (29.875 ± 4.342) (P = 0.7956), while the HPC-AH group showed significantly higher spontaneous alternation (P<0.05) (Fig. 5E). In the Y-maze novel arm exploration test (Fig. 5F), the total entries remained comparable between groups AH: 30.75 ± 3.666, vs. HPC-AH: 31 ± 3.606, P = 0.8995), while the percentage of entries into the new arm was higher in the HPC-AH group than in the AH group. (*P*<0.01) (Fig. 5G).

**Figure 6.**
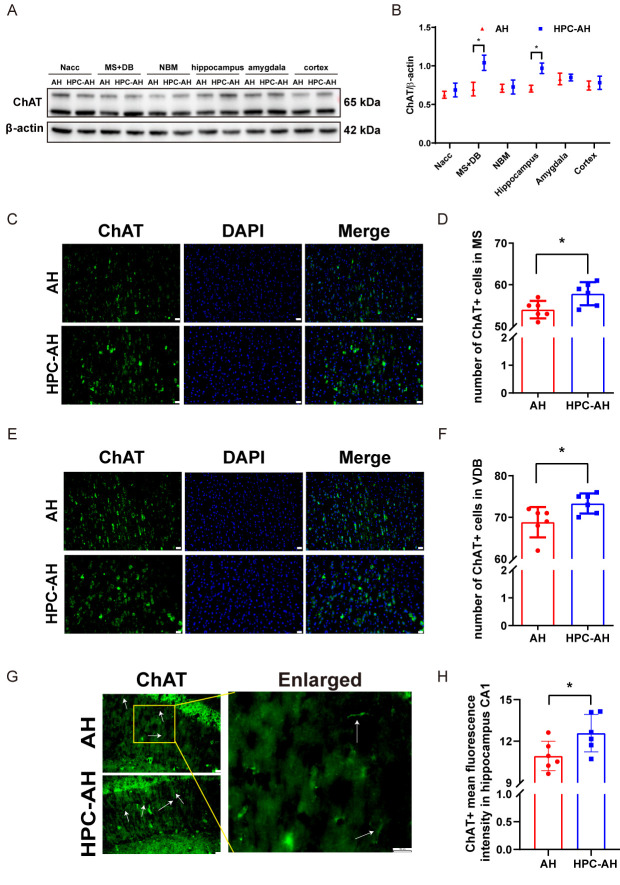
**HPC mitigates AH-induced cholinergic neuronal cell loss in MS/VDB**. (A, B) Western blot analysis of ChAT expression in various nuclei, including representative blot images and quantitative analysis. (C, D) Representative images and quantified number of ChAT-immunopositive cells per frame in the MS (n=6). (E, F) Representative images and quantified number of ChAT-immunopositive cells per frame in the VDB (n=6). (G, H) Representative images and quantified ChAT-immunopositive mean fluorescence intensity (showing cholinergic fibers (arrow heads) per frame in the hippocampus CA1. (n=6). Data are shown as mean±SD. Significant differences as determined by unpaired Student’s t-test (two-tailed). *P < 0.05, **P < 0.01, unpaired t-test. Scale bars, 20 µm.

**Figure 7.**
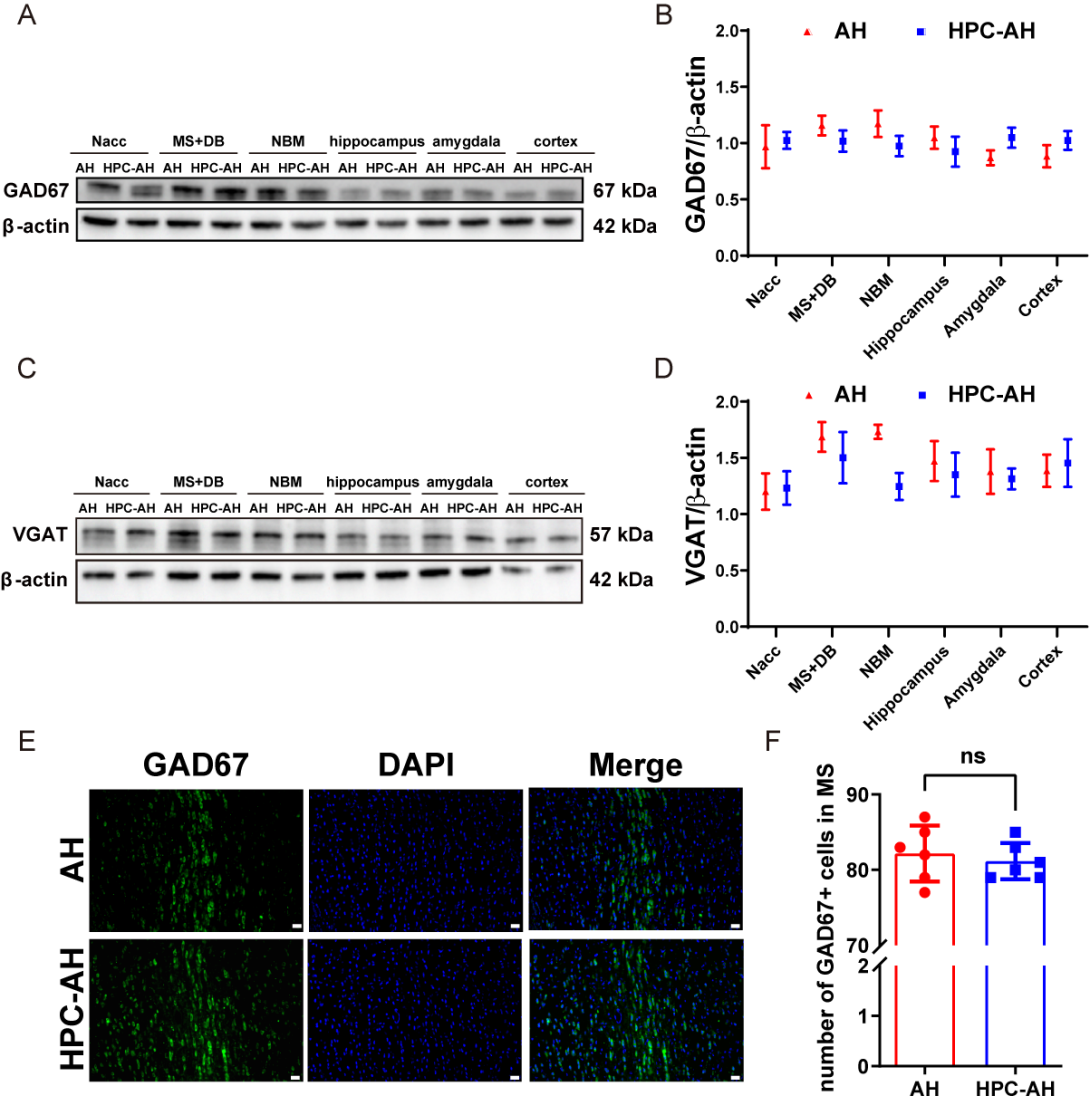
**HPC shows no significant effect on MS GABAergic neurons in mice**. (A, B) Western blot analysis of GAD67 expression in various nuclei, including representative blot images and quantitative analysis (n=5). (C, D) Western blot analysis of VGAT expression in various nuclei, including representative western blot images and quantitative analysis (n=5). (E, F) Representative images and quantified number of GAD67-immunopositive cells per frame in the MS (n=6). Data are shown as mean±SD. Significant differences as determined by unpaired Student’s t-test (two-tailed). ns: no significant differences, unpaired t-test. Scale bars, 20 µm.

In the cued fear conditioning and extinction test (Fig. 5H), freezing levels at baseline (Days 1 and 2) were comparable between the HPC-AH and AH groups (P > 0.05 for both days; Fig. 5I, 5J). Following conditioning shocks on Day 1, within-group analysis revealed that the HPC-AH group exhibited a significant increase in freezing levels on Day 2 compared to baseline, indicating successful fear acquisition. Between-group comparisons further showed that the HPC-AH group had higher freezing levels than the AH group during the first tone presentation on Day 2.

**Figure 8.**
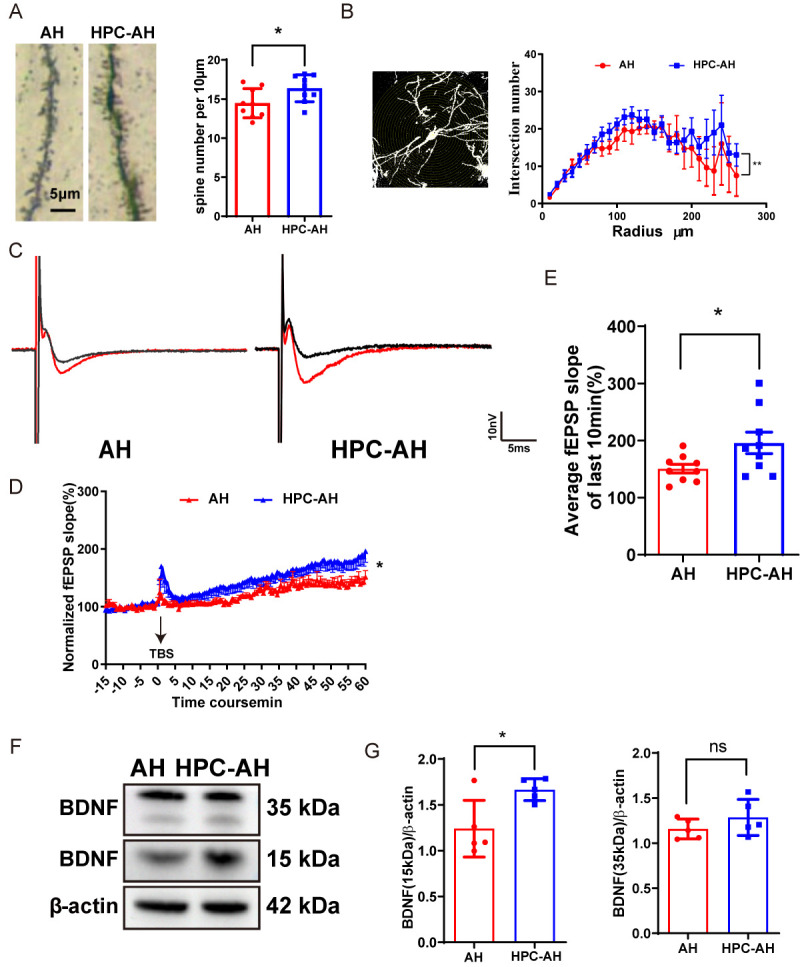
**HPC alleviates AH-induced hippocampal synaptic dysfunction**. (**A**) Representative Golgi staining images showing the dendritic spines, scale bars = 5μm, and number of dendritic spines per 10 μm (n=9). (**B**) Representative images about hippocampal neuron sholl analysis. (**C**) Representative traces of fEPSP before (black curve) and after (red curve) the stimulation of TBS in LTP induction. (**D**) Time course changes in normalized fEPSP slopes (%). fEPSP were induced by TBS. (**E**) Quantitative analysis of average fEPSP slopes in the last 10 min of LTP (n=9/6). (F, G) Western blot analysis of BDNF expression of hippocampus, including representative blot images and quantitative analysis (n=5). Data are shown as mean±SD. Significant differences as determined by unpaired Student’s t-test (two-tailed). ns: no significant differences; *P < 0.05, unpaired t-test and repeated-measures ANOVA analysis of variance.

On Day 3, within the HPC-AH group, freezing levels significantly decreased across trials, reflecting successful extinction retention. In contrast, the AH group showed no temporal decline in freezing. Between-group comparisons demonstrated that the HPC-AH group exhibited lower freezing levels than the AH group by the final trial (P < 0.001) (Fig. 5K). These results collectively indicate that HPC restores both fear acquisition and extinction retention disrupted by AH.

The open field test showed no differences in locomotor activity between the groups (Fig. 5L-O) The novel object recognition test indicated improved cognitive function in the HPC-AH group, with a significantly reduced RI (*P*<0.05) (Fig. 5P, Q). These results indicated that HPC-AH improves hippocampus-related cognitive dysfunction caused by AH exposure without affecting cortex-related cognitive function and locomotor function in mice. This finding is consistent with our above results on cognitive dysfunction caused by AH.

### HPC mitigates AH-induced cholinergic cell loss in MS/VDB

To determine whether HPC ameliorates AH-induced cholinergic cell damage in the MS/VDB, we examined the survival of cholinergic cells within individual nuclei 5 days after HPC. Western blot experiments showed that HPC-AH increased ChAT expression in the MS/VDB and hippocampus (*P*<0.05) (Fig. 6A, B). Immunofluorescence staining further revealed a significant increase in cholinergic cells in MS/VDB and cholinergic fiber intensity in hippocampal CA1 region (Fig. 6C-H). These findings suggest that HPC ameliorates cholinergic cell damage in MS/VDB and hippocampal areas of AH mice.

To determine whether HPC affects GABAergic cells in the MS and hippocampal regions of AH mice, we used GAD67 and VGAT as markers. Western blot and immunofluorescence staining experiments showed no significant changes in the expression of GAD67 or VGAT (Fig. 7A-D), nor in the number of GABAergic cells (Fig. 7E-F). These findings suggest that HPC has no effect on GABAergic neurons in the MS and hippocampal areas of AH mice.

### HPC alleviates AH-induced hippocampal synaptic dysfunction

Changes in synaptic functions are generally accompanied by changes in synaptic density. Thus, we performed Golgi staining in the CA1 region of the hippocampus to observe the density of dendritic spines of AH and HPC-AH mice. We found that HPC treatment improved the density of dendritic spines in hippocampal CA1 of AH group mice (*P*<0.0001) (Fig. 8A). HPC significantly increases the complexity of hippocampal neurons by sholl analysis (Fig. 8B). Additionally, to investigate whether HPC ameliorates AH impairment of synaptic plasticity in the mice hippocampus, we performed electrophysiological recordings to assess LTP. The results showed that while the AH group maintained LTP, HPC improved its attenuation (Fig. 8C-E). Using Western blot analysis, we found that HPC increased the expression of BDNF (15kDa) in AH mice (Fig. 8F, G).

## DISCUSSION

In the present study, we demonstrated for the first time that AH induces cholinergic cell loss in the medial septal nucleus (MS) and hippocampus, consequently resulting in cognitive dysfunction. Our findings indicate that AH not only impairs synaptic function, but also reveals that the disruptions of the projections from the MS to the hippocampus, which may be a key factor underlying the AH-induced cognitive impairments. Our study also found that HPC can alleviate these adverse effects by protecting the survival of cholinergic cells and improving hippocampal synaptic plasticity. This finding provides novel insights into the mechanisms by which HPC confers neuroprotection against AH, particularly in the prevention and treatment of cognitive dysfunction induced by AH.

Emerging studies indicate a link between AH and cognitive impairment [5, 51, 52]. One study demonstrated that mice exhibited cognitive impairments under simulated conditions of 8,000 meters (8% O_2_) on Day 1, also showed cognitive deficits at simulated altitudes of 6,000 meters on Days 3 and 7 [53]. Additionally, another study reported that mice exposed to simulated 6,000-meter hypobaric hypoxia (10% oxygen) for 7 days exhibited significant cognitive impairments but no locomotor deficits[54]. Importantly, the absence of significant differences in total Y-maze entries between groups suggests that the observed cognitive impairments were not secondary to reduced locomotor activity, further supporting the specificity of hypoxia-induced deficits in hippocampal and cholinergic function. These findings are consistent with our results, indicating that AH affects cognitive function but not locomotor function in mice. However, the mechanisms behind this association remain unclear. Recent research has primarily focused on oxidative stress and inflammation as contributing factors [55, 56]. Nonetheless, the potential critical role of disrupted projections between nuclei in memory impairments caused by AH has not been fully explored. It is well-established that dysfunctional neural circuits can cause cognitive impairment [57-59], suggesting a complex interplay of factors contributing to the observed cognitive deficits.

Additionally, accumulating evidence suggests that the neurotransmitter, including both excitatory and inhibitory neurotransmitters dysregulation, may cause interneuron dysfunction, contributing to cognitive deficits [60]. Key neurotransmitters such as Acetylcholine and GABA play crucial roles in this process. GABA is the predominant inhibitory neurotransmitter in the brain, and an imbalance of GABA neurons leads to neurological dysfunction [61, 62]. Acetylcholine, derived from cholinergic cells, is an important excitatory neurotransmitter that is closely related to nervous system functions. Cholinergic neurons originate in the MS and DB regions of the basal forebrain and project to nuclei in the cortex, hippocampus, and amygdala [63, 64]. The extensive and complex fiber projection of cholinergic neurons form the cholinergic neural circuits, which modulate several nuclei in the brain through neurotransmission and are involved in learning, memory, attention, emotion, and locomotion. The loss and degeneration of cholinergic neurons or abnormal theta oscillations and cholinergic neural circuits induces cognitive dysfunction such as Alzheimer's disease, cerebral ischemia, and Parkinson's disease [65-67]. Specifically, the loss of cholinergic cell between basal forebrain and hippocampus correlates highly with the memory impairments [68]. Furthermore, studies have found that BF cholinergic input to the ventromedial prefrontal cortex and hippocampus is critical for neural function in these regions, suggesting that BF cholinergic neurons may be critical for fear and extinction memory [39, 41, 69]. In our study, the observed correlations between MS/VDB cholinergic cell loss and fear acquisition deficits (Fig. 2M, N) align with the established role of basal forebrain cholinergic neurons in fear memory circuits. The positive association between hippocampal CA1 cholinergic fiber density and performance in novel object recognition and Y-maze spontaneous alternation tasks (Fig. 2O, P) underscores the importance of hippocampal cholinergic innervation in spatial and recognition memory. Acetylcholine enhances CA1 pyramidal neuron excitability and theta rhythm generation, which are essential for LTP and memory consolidation [70, 71]. The lack of correlation with novel arm exploration may reflect the distinct neural circuits involved in spatial novelty detection, which rely more on hippocampal glutamatergic signaling and cortical integration[32]. These findings collectively highlight region-specific contributions of cholinergic pathways to different cognitive domains under hypoxic stress.

Although the hippocampus is well-documented as highly susceptible to hypoxia-induced damage[72], our study found no significant neuronal loss in the hippocampal CA1 region following AH exposure. This contrasts with previous findings and may be explained by the relatively short duration and less severe hypoxia used in our study (3 days at 8% O_2_), which might not have been sufficient to induce neuronal death. Additionally, the observed cognitive deficits may also be driven by the loss of cholinergic inputs from the medial septum and vertical diagonal band, which are crucial for hippocampal function, rather than direct neuronal death. In our study, AH impaired fear extinction while leading to cholinergic cell loss in MS/VDB (Fig. 2H-J). Since the fear extinction task measures MS/VDB function [39], we speculate that the observed cognitive deficits may stem from cholinergic cell loss in the MS/DB-hippocampus rather than direct neuronal death in the hippocampus. This is supported by our finding that, despite cognitive impairments, there was no significant neuronal loss in the hippocampal CA1 region following AH exposure (3 days at 8% O_2_). This contrasts with previous studies showing substantial hippocampal damage under more prolonged or severe hypoxic conditions[72]. The discrepancy may be explained by the relatively short duration and less severe hypoxia used in our study, which might not have been sufficient to induce neuronal death. Additionally, the loss of cholinergic projections from the MS and VDB could impair hippocampal function without causing significant neuronal loss. Cholinergic neurons in the MS provide major projections to the hippocampus, influencing changes in excitatory synaptic transmission and induces hippocampal theta oscillatory activity [73].

In the hippocampus, cholinergic activity modulates neuronal excitability, network activity, as well as synaptic transmission and plasticity [70, 71]. In the CA1 region, ACh induces CA1 pyramidal neuron depolarization, theta rhythm generation, and LTP of glutamatergic CA3-CA1 synaptic transmission [71, 74, 75]. Notably, in our study, we found AH does not appear to induce hippocampal neuron loss (Fig. 4D-G). Although the mechanisms underlying these effects are unknown, our current findings implicate MS cholinergic cell loss in the manifestation of AH-induced hippocampal synaptic dysfunction.

Cholinergic cells in the MS/DB are critical for regulating/generating hippocampal theta rhythm, which has been shown to facilitate LTP processes and play important roles in spatial learning and memory [43, 76]. As MS cholinergic lesions reduce hippocampal theta activity [76], we speculate that AH induce potential hippocampal theta rhythm, then result in LTP impairments. In addition, our morphological analysis revealed that there is a decreased proportion of mature spines in AH mice. It is important to highlight that dendritic spines represent the main unitary postsynaptic compartment for excitatory input, the basis for LTP induction [77]. In other words, AH results in deficient cholinergic innervation and impaired dendritic spine maturation in the hippocampus where cholinergic neurons project, thereby results in impaired long-term potentiation and cognitive dysfunction in mice. We found that AH decreased the expression of BDNF in hippocampus compared to the sham group. BDNF is an important regulator of synaptic transmission and LTP in the hippocampus and in other brain regions, playing a role in the formation of certain forms of memory [78]. Specifically, BDNF promotes the growth and maturation of dendritic spines, which are essential for excitatory synaptic transmission and LTP [37]. The decrease in BDNF expression may consequently decrease dendritic spine density and impair synaptic plasticity [37], as demonstrated by LTP in the hippocampal CA1 region (Fig. 4A-C). This finding underscores the importance of BDNF in maintaining cognitive function under hypoxic conditions.

Cognitive dysfunction induced by AH creates a great burden on daily living and work. There is an urgent need for strategies to address or prevent the suffering caused by AH. Multiple studies found that the HPC attenuates injury during high-altitude exposure [79, 80]. It has also been found in animal studies that HPC improves chronic hypoxia-induced cognitive dysfunction [26]. However, its function in AH has not yet been well studied. In our study, we found that 5 days of HPC prior to AH significantly improved AH-induced cognitive dysfunction. Importantly, hypoxic preconditioning also attenuated the loss of cholinergic cells in the MS/VDB and hippocampal regions and improved synaptic dysfunction. Similarly, we also demonstrated that HPC was able to rescue the reduction of BDNF brought about by AH.

Although our findings provide novel insight into mechanisms underlying HPC-mediated neuroprotection against AH, some major limitations need to be addressed. First, we do not show direct evidence that MS/VDB cholinergic cell loss is responsible for the observed deficits in hippocampal LTP following AH. Second, we also lack direct evidence implicating cholinergic cell loss as the primary target of HPC-mediated rescue of hippocampal LTP impairments. Ablation of MS/VDB cholinergic function in the presence of HPC after AH is required to elucidate a direct link and will be addressed in future studies. Third, our analysis focused primarily on cholinergic cell loss in the MS/VDB and hippocampus, while changes in cholinergic fibers projecting to other brain regions (e.g., the cortex or amygdala) were not systematically investigated. Although our Western blot data indicated no significant alterations in ChAT expression in cortical regions under AH (Fig. 2B), a comprehensive assessment of cholinergic fiber density and functional connectivity across multiple brain areas is warranted. Additionally, our study used only young adult male mice (8 weeks old), limiting the translational relevance to older individuals and ignoring sex differences in hypoxia susceptibility and cognitive function. Future research should explore the effects of HPC in older cohorts and include both sexes to better understand the mechanisms involved. Future studies should employ advanced techniques such as whole-brain imaging or circuit-specific tracing to evaluate how hypoxia disrupts broader cholinergic networks. Multi-regional joint analyses would further clarify whether cognitive deficits arise from localized hippocampal dysfunction or global network imbalances, thereby refining targeted therapeutic strategies. In summary, the present study reveals the impact of AH on cholinergic neurons and cognitive function and demonstrates the potential of HPC in alleviating these effects. These findings provide an important theoretical basis for the future development of therapeutic strategies targeting cognitive dysfunction induced by AH.

Our findings hold translational potential for both environmental and clinical hypoxic scenarios. For instance, HPC protocols could mitigate cognitive deficits in high-altitude travelers or military personnel [79, 80] Additionally, in clinical contexts such as stroke or cardiac arrest, HPC may complement existing therapies by preserving cholinergic integrity and enhancing BDNF signaling [67, 72, 81].

Future studies should focus on optimizing HPC protocols (like duration, frequency, and oxygen levels) for human applicability and evaluating its efficacy in clinical cohorts. Moreover, combining HPC with pharmacological agents targeting cholinergic pathways or BDNF signaling may synergistically enhance neuroprotection in AH scenarios.

## Data Availability

Data will be made available on request.
